# Environmental Temperature Variation Affects Brain Lipid Composition in Adult Zebrafish (*Danio rerio*)

**DOI:** 10.3390/ijms25179629

**Published:** 2024-09-05

**Authors:** Elisa Maffioli, Simona Nonnis, Armando Negri, Manuela Fontana, Flavia Frabetti, Anna Rita Rossi, Gabriella Tedeschi, Mattia Toni

**Affiliations:** 1 Department of Veterinary Medicine and Animal Science (DIVAS), Università degli Studi di Milano, Via dell’Università 6, 26900 Lodi, Italy; elisa.maffioli@unimi.it (E.M.); simona.nonnis@unimi.it (S.N.); armando.negri@unimi.it (A.N.); 2 CRC “Innovation for Well-Being and Environment” (I-WE), Università degli Studi di Milano, 20126 Milano, Italy; 3 Unitech OMICs, Università degli Studi di Milano, 20139 Milan, Italy; manuela.fontana@unimi.it; 4 Department of Medical and Surgical Sciences—DIMEC, University of Bologna, 40126 Bologna, Italy; flavia.frabetti@unibo.it; 5 Department of Biology and Biotechnologies “Charles Darwin”, Sapienza University, 00185 Rome, Italy

**Keywords:** lipid, brain, temperature, zebrafish

## Abstract

This study delves deeper into the impact of environmental temperature variations on the nervous system in teleost fish. Previous research has demonstrated that exposing adult zebrafish (*Danio rerio*) to 18 °C and 34 °C for 4 or 21 days induces behavioural changes compared to fish kept at a control temperature of 26 °C, suggesting alterations in the nervous system. Subsequent studies revealed that these temperature conditions also modify brain protein expression, indicating potential neurotoxic effects. The primary aim of this work was to investigate the effects of prolonged exposure (21 days) to 18 °C or 34 °C on the brain lipidomes of adult zebrafish compared to a control temperature. Analysis of the brain lipidome highlighted significant alteration in the relative abundances of specific lipid molecules at 18 °C and 34 °C, confirming distinct effects induced by both tested temperatures. Exposure to 18 °C resulted in an increase in levels of phospholipids, such as phosphatidylethanolamine, alongside a general reduction in levels of sphingolipids, including sphingomyelin. Conversely, exposure to 34 °C produced more pronounced effects, with increases in levels of phosphatidylethanolamine and those of various sphingolipids such as ceramide, gangliosides, and sphingomyelin, alongside a reduction in levels of ether phospholipids, including lysophosphatidylethanolamine ether, phosphatidylethanolamine ether, and phosphatidylglycerol ether, as well as levels of glycolipids like monogalactosyldiacylglycerol. These results, when integrated with existing proteomic and behavioural data, offer new insights into the effects of thermal variations on the nervous system in teleost fish. Specifically, our proteomic and lipidomic findings suggest that elevated temperatures may disrupt mitochondrial function, increase neuronal susceptibility to oxidative stress and cytotoxicity, alter axonal myelination, impair nerve impulse transmission, hinder synapse function and neurotransmitter release, and potentially lead to increased neuronal death. These findings are particularly relevant in the fields of cell biology, neurobiology, and ecotoxicology, especially in the context of global warming.

## 1. Introduction

Environmental conditions significantly impact organism survival, with variation in biotic and abiotic factors presenting fundamental challenges. Temperature, a critical abiotic factor, profoundly influences both homeothermic and ectothermic organisms, affecting their physiology, behaviour, population size, and geographic distribution [1]. Ectothermic organisms, such as fish, are particularly sensitive to thermal fluctuations in their environments due to their inability to regulate their internal temperature.

Their ecological response to such changes is primarily behavioural, involving movement to more favourable environments [2]. However, when relocation is not feasible, fish must acclimatise and adapt through complex physiological processes, including temperature compensation, to survive [3]. In such situations, even slight external temperature changes can impact cells and tissues, including the central nervous system (CNS), which controls an organism’s functions and behaviour.

This adaptation involves various organs and cellular processes, such as transcription and translation regulation, enabling the production of temperature-specific isoenzymes [4,5], the synthesis of molecular chaperones [6,7], the alteration of membrane lipid components [8], and changes in mitochondrial density and characteristics [9,10].

Understanding the effects of thermal alterations is crucial from both an ecological perspective, especially in the context of global warming, and a physiological perspective to comprehend cellular response to stress [11]. Given its complexity, studying the impact of specific treatments on the CNS requires a multidisciplinary approach, integrating omics techniques such as genomics, transcriptomics, proteomics, metabolomics, lipidomics [12,13,14], and behavioural assessments [15].

Recent studies conducted by our research group have shown that thermal changes within an organism’s survival range can affect the nervous systems of teleost fish [16,17,18,19,20,21,22,23].

Among teleosts, the zebrafish serves as an excellent model organism widely utilised across various research fields, including neuroscience, where omics approaches [24] and validated behavioural tests [25] are often applied. 

Zebrafish are particularly well-suited for studying the effects of thermal changes due to their broad thermal tolerance, characterised by a viability range estimated to span from 6 °C to 43 °C and a range of tolerable temperatures from 4.5 °C to 42 °C [1,26,27]. Additionally, the zebrafish’s natural habitat in Asia [28] is marked by daily temperature fluctuations of approximately 5 °C and significant seasonal variations ranging from 6 °C in the winter to over 38 °C in the summer [29]. Zebrafish particularly prefer brooks, lakes, ponds, and rice fields [30,31], where temperatures generally vary from 16.5 °C to 34.0 °C [32]. This makes the zebrafish an ideal model for investigating ecologically relevant temperature variations within its tolerance range [33].

Our research group has previously explored the effects of thermal changes on the nervous system by exposing adult zebrafish to 18 °C (the low-temperature treatment), 26 °C (the control), or 34 °C (the high-temperature treatment) for 4 days (acute exposure) or 21 days (chronic exposure). These temperature conditions were selected based on Vergauwen et al.’s work [34], as they fall within the zebrafish’s vital range and reflect temperatures encountered in their natural environment. The control temperature was set to 26 °C because this corresponds to the thermal preferendum of zebrafish [26].

Behavioural tests, including novel tank diving, light and dark preferences, social preferences, and mirror biting, have revealed significant behavioural changes in zebrafish exposed to 18 °C or 34 °C for 4 or 21 days [20,22,23]. Specifically, low temperatures induced anxiety-like behaviours, while high temperatures reduced anxiety and increased boldness. Moreover, the Y-maze test suggested that low and high temperatures might alter cognitive abilities [19,20,22]. Subsequent analysis of the brain proteome under these thermal conditions confirmed alterations in the nervous system, revealing changes in the expression of several proteins involved in critical cellular processes [19,20], indicative of a neurotoxic effect due to thermal alteration [18,19,20,21,22,23].

Scientific evidence highlights brain membranes, including both plasma and cellular organelle membranes, as key targets of thermal variation, necessitating alteration in lipid components for variable homeoviscous responses [35,36,37,38].

Building on the robust foundation of behavioural and proteomic research on zebrafish conducted by our group over the past five years, this study expands our understanding of thermal variation effects by analysing how 21-day exposure to 18 °C, 26 °C, and 34 °C impacts brain lipid composition in adult zebrafish. Investigating thermal variation’s impact on brain lipid composition is crucial for comprehending thermal adaptation mechanisms in the CNS, as lipids are vital for various cellular functions, including membrane formation, intercellular signalling, energy storage, and homeostasis maintenance [14].

## 2. Results

**Figure 1:**
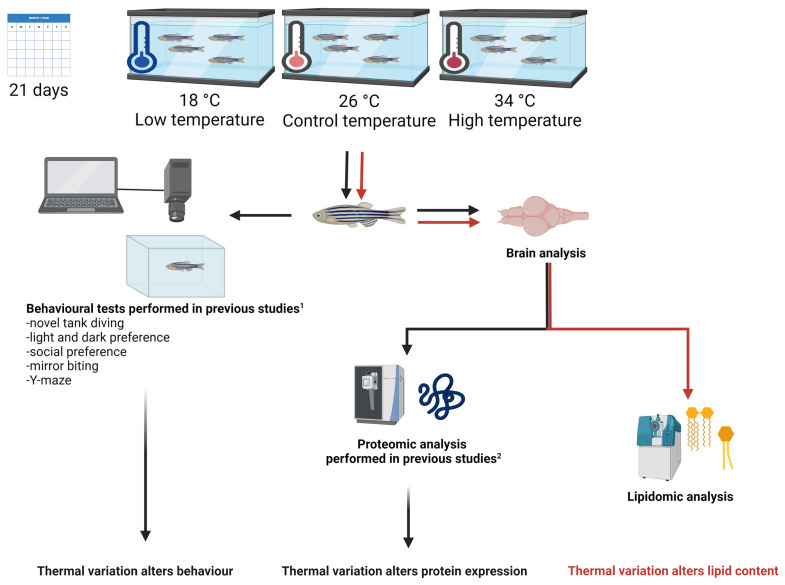
Experimental framework for analysing thermal variation effects in adult zebrafish. Fish were maintained at 18 °C (low temperature), 26 °C (control temperature), or 34 °C (high temperature) for 21 days (chronic exposure). After their treatment, the fish were euthanised, and their brains were surgically dissected for lipidomic analysis (red line and arrows). Previous studies conducted by our research group investigated behavioural and brain proteomic responses in zebrafish exposed to the same thermal conditions (black line and arrows). The results indicate that thermal variation significantly alters behaviour, protein expression, and lipid content in the zebrafish brain. ^1^ and ^2^ refer to the bibliographic citations of previous works [19,20,21,22,23].

The brains of adult zebrafish were collected from specimens subjected to three temperature conditions, namely, 18 °C, 26 °C (control), and 34 °C, for 21 days and analysed via untargeted and targeted lipidomic approaches for the identification and quantification of different lipid classes and gangliosides, respectively (Figure 1).

**Figure 2:**
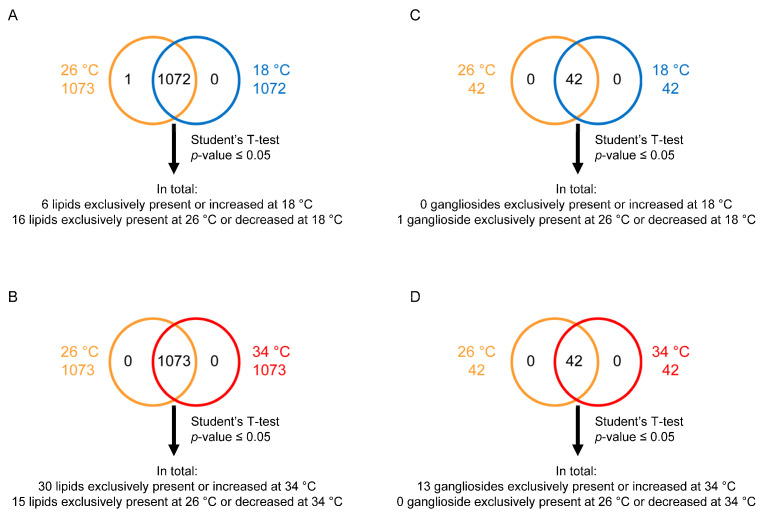
Extraction and analysis of zebrafish brain lipids. Venn diagrams depicting lipids extracted from brains of adult zebrafish subjected for 21 days to three temperature conditions, namely, 18 °C, 26 °C (control), and 34 °C, and analysed via untargeted (**A**,**B**) and targeted lipidomics approaches (**C**,**D**). A statistical evaluation was applied for each one-to-one comparison. Lipids were considered to be differentially expressed if they were present only in one condition or showed a significant *t*-test difference (Student’s *t*-test *p*-value  ≤  0.05).

In the present study, the untargeted analysis allowed identifying 1073 lipids at both 26 °C and 34 °C and 1072 lipids at 18 °C, as reported in Supplementary Table S1. To disclose the influence of temperature exposure on the zebrafish’s brain lipidome, specific analyses were carried out via one-to-one comparison: 18 °C vs. 26 °C and 34° vs. 26 °C. According to the results, the levels of 6 lipids increased at 18 °C, the levels of 15 lipids decreased at 18 °C, and 1 lipid was present only at 26 °C in the 18 °C vs. 26 °C comparison (*p*-value ≤ 0.05) (Supplementary Table S2, Figure 2A). Concerning the comparison of 34° with 26 °C (*p*-value ≤ 0.05), the levels of 30 lipids were increased at 34 °C and those of 15 lipids were decreased (Supplementary Table S3, Figure 2B).

The targeted analysis allowed identifying gangliosides at 26 °C, 34 °C, and 18 °C (Supplementary Table S4), among which the levels of 1 were decreased at 18 °C in the 18 °C vs. 26 °C comparison and the levels of 13 were increased at 34 °C in the 34 °C vs. 26 °C comparison (*p*-value ≤ 0.05) (Supplementary Table S5, Figure 2C,D). Overall, the results of these comparisons reveal that temperatures of 18 and 34 °C altered the lipid profiles of the corresponding zebrafish compared to the control (26 °C), prompting further analysis to better understand the effect of the thermal stress.

**Figure 3:**
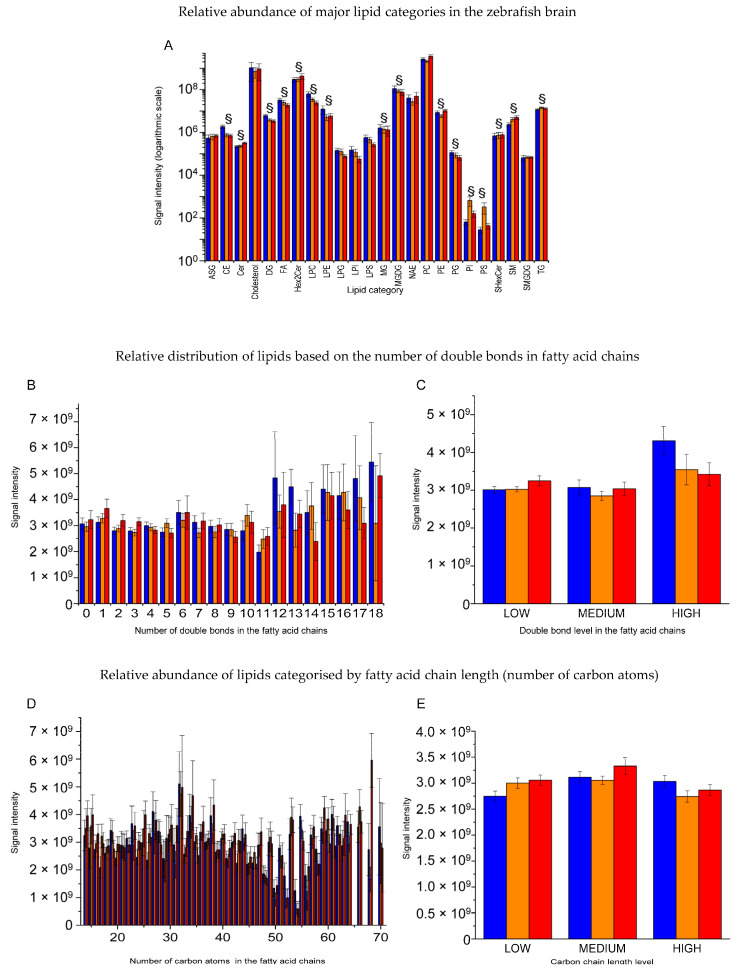
Untargeted brain lipid profile of adult zebrafish maintained at temperatures of 18 °C (blue), 26 °C (orange), and 34 °C (red). (**A**): relative amount of the main lipid categories represented with a logarithmic scale. Analysis with two-way ANOVA followed by Tukey’s post hoc test only identified significant differences between phosphatidylcholine and other lipid categories (§, *p* ≤ 0.05); (**B**): relative quantity of lipids with different numbers of double covalent bonds in the fatty acid chains. (**C**): relative quantity of lipids characterised by low (0–6), medium (7–12), and high (13–18) numbers of double covalent bonds. Analysis with two-way ANOVA followed by Tukey’s post hoc test did not identify significant differences in the content of double covalent bonds between temperatures. (**D**): relative quantity of lipids characterised by different sizes evaluated by the number of carbon atoms in the fatty acid chains. (**E**): relative quantity of lipids characterised by low (0–25), medium (26–50), and high (51–75) numbers of carbon atoms. Analysis with two-way ANOVA followed by Tukey’s post hoc test did not identify significant differences in the content of double covalent bonds between temperatures. The data are expressed as means ± S.E.M. in all panels. ASG (acylhexose glutathione); CE (cholesterol ester); Cer (ceramides); Cholesterol (cholesterol); DG (diacylglycerol); FA (fatty acid); Hex2Cer (dihexosyl-ceramides); LPC (lysophosphatidylcholine); LPE (lysophosphatidylethanolamine); LPG (lysophosphatidylglycerol); LPI (lysophosphatidylinositol); LPS (lysophosphatidylserine); MG (monoglyceride); MGDG (monogalactosyldiacylglycerol); NAE (N-acylethanolamine); PC (phosphatidylcholine); PE (phosphatidylethanolamine); PG (phosphatidylglycerol); PI (phosphatidylinositol); PS (phosphatidylserine); SHexCer (sulfatides/sulfated hexosyl ceramide); SM (sphingomyelin); SMGDG (sulfomonohexosyldiacylglycerol); TG (triacylglycerol).

To investigate whether exposure to 18 °C or 34 °C resulted in significant alterations in major lipid classes compared to the control temperature, the untargeted lipidomics data set (Table S1) was analysed using a two-way ANOVA, focusing on the factors lipid categories and temperature. This analysis revealed significant differences between lipid categories [F(23, 12,804) = 22.407, *p* < 0.001] but no significant effect of temperature [F(2, 12,804) = 2.552, *p* = 0.078] and no interaction effect between temperature and lipid categories [F(46, 12,804) = 0.578, *p* = 0.990]. The results of Tukey’s post hoc test confirmed that phosphatidylcholines (PCs) were significantly more abundant than the other lipid categories (Figure 3A). 

Given that the fluidity of lipid membranes can be influenced by the level of unsaturation, i.e., the number of double covalent bonds in the fatty acid chains and the length of the carbon chain, the untargeted lipidomic data set (Table S1) was further analysed via a two-way ANOVA considering the factors temperature and size of lipid molecules, as well as temperature and unsaturation level. This analysis showed no significant differences in the overall lipid unsaturation level across the three temperatures [F(36, 12,807) = 0.141, *p* = 1.000], although there was a trend towards an increase in levels of unsaturated lipids at 18 °C (Figure 3B,C). Additionally, no significant differences in the sizes of the molecules across the three temperatures were observed [F(106, 12,702) = 0.084, *p* = 1.000] (Figure 3D,E).

### 2.1. Lipids Were Significantly Altered at 18 °C Compared to 26 °C

**Figure 4:**
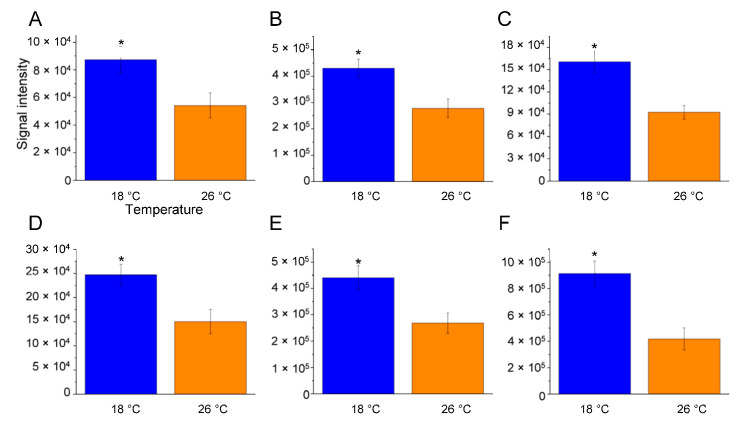
Lipid levels significantly higher at 18 °C compared to 26 °C. Bar graphs show the relative amounts of specific brain lipids at 18 °C (blue) and 26 °C (orange), focusing on those significantly elevated in the 18 °C vs. 26 °C comparison. Lipid species are as follows: (**A**): PE 36:3;O|PE 16:0_20:3;O (C41H76NO9P); (**B**): PE 38:3;O|PE 18:0_20:3;O (C43H80NO9P); (**C**): PE 40:4;O|PE 18:1_22:3;O (C45H82NO9P); (**D**): PE 42:9;O|PE 22:6_20:3;O (C47H76NO9P); (**E**): PE 44:9;O|PE 22:6_22:3;O (C49H80NO9P); (**F**): SM 40:7;O3 (C45H79N2O7P). The data are expressed as means ± S.E.M. and were analysed using one-way ANOVA with Tukey’s post hoc correction. * *p* ≤ 0.05 in 18 °C versus 26 °C comparison. *p*-values are reported in Table 1. *N* = 4.

To identify specific lipid molecules that exhibited significant differences in relative abundance when comparing the 18 °C vs. 26 °C or 34 °C vs. 26° C comparisons, a detailed analysis of both untargeted and targeted datasets was conducted on the single lipid molecules via one-way ANOVA focusing on temperature as the primary factor. In cold-acclimatised zebrafish, a significant increase in the levels of certain phospholipid molecules was observed compared to those at the control temperature (Table 1, Figure 4). Specifically, several phosphatidylethanolamine (PE) molecules with three to nine double covalent bonds, such as PE 36:3;O|PE 16:0_20:3;O (*p* < 0.0437), PE 38:3;O|PE 18:0_20:3;O (*p* < 0.0152), PE 40:4;O|PE 18:1_22:3;O (*p* < 0.0040), PE 42:9;O|PE 22:6_20:3;O (*p* < 0.0184), and PE 44:9;O|PE 22:6_22:3;O (*p* < 0.0230) (Figure 4A–E), showed significant increases at 18 °C.

**Figure 5:**
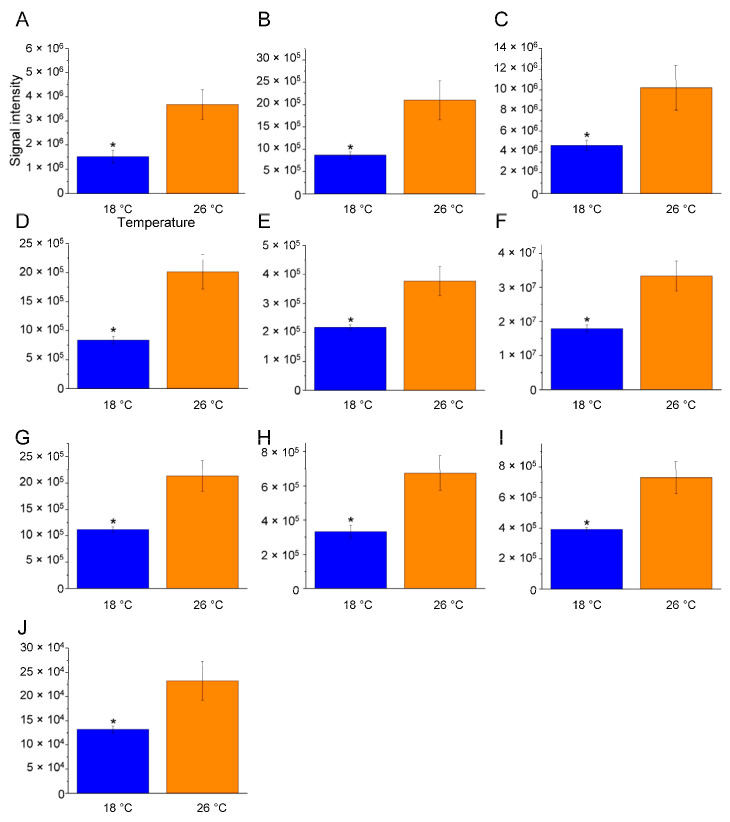
Lipid levels significantly reduced at 18 °C compared to 26 °C. Bar graphs display the relative amounts of brain lipids at 18 °C (blue) and 26 °C (orange), focusing on those significantly decreased in the 18 °C vs. 26 °C comparison. Lipid species are as follows: (**A**): SM 37:3;O2 | SM 19:3;O2/18:0 (C42H81N2O6P); (**B**): SM 38:1;O2 | SM 21:0;O2/17:1 (C43H87N2O6P); (**C**): SM 40:1;O2 | SM 18:1;O2/22:0 (C45H91N2O6P); (**D**): SM 40:2;O2 | SM 18:1;O2/22:1 (C45H89N2O6P); (**E**): SM 42:1;O3 (C47H95N2O7P); (**F**): SM 42:2;O2 | SM 18:1;O2/24:1 (C47H93N2O6P); (**G**): SM 42:3;O2 | SM 18:1;O2/24:2 (C47H91N2O6P); (**H**): SM 42:4;O2 | SM 18:1;O2/24:3 (C47H89N2O6P); (**I**): SM 44:3;O2 | SM 18:1;O2/26:2 (C49H95N2O6P); (**J**): SM 44:4;O2 | SM 18:1;O2/26:3 (C49H93N2O6P). The data are expressed as means ± S.E.M. and were analysed via one-way ANOVA with Tukey’s post hoc correction. * *p* ≤ 0.05 in 18 °C versus 26 °C comparison. *p*-values are reported in Table 1. *N* = 4.

Conversely, a general reduction in sphingolipid composition was noted in the cold-acclimatised zebrafish. The levels of several sphingomyelin (SM) molecules, characterised by fatty acid chains containing 37 to 44 carbon atoms and one to four double bonds, were reduced at 18 °C (Table 1 and Figure 5). Notably, SM 37:3;O2|SM 19:3;O2/18:0 (*p* < 0.0248), SM 38:1;O2|SM 21:0;O2/17:1 (*p* < 0.0253), SM 40:1;O2|SM 18:1;O2/22:0 (*p* < 0.0442), SM 40:2;O2|SM 18:1;O2/22:1 (*p* < 0.0071), SM 42:1;O3 (*p* < 0.0219), SM 42:2;O2|SM 18:1;O2/24:1 (*p* < 0.0188), SM 42:3;O2|SM 18:1;O2/24:2 (*p* < 0.0144), SM 42:4;O2|SM 18:1;O2/24:3 (*p* < 0.0216), SM 44:3;O2|SM 18:1;O2/26:2 (*p* < 0.0256), and SM 44:4;O2|SM 18:1;O2/26:3 (*p* < 0.0497) (Figure 5A–J) showed a significant decrease, while only SM 40:7;O3 (*p* < 0.0101) increased at 18 °C compared to 26 °C (Figure 4F).

### 2.2. Lipids Significantly Altered at 34 °C Compared to 26 °C

**Figure 6:**
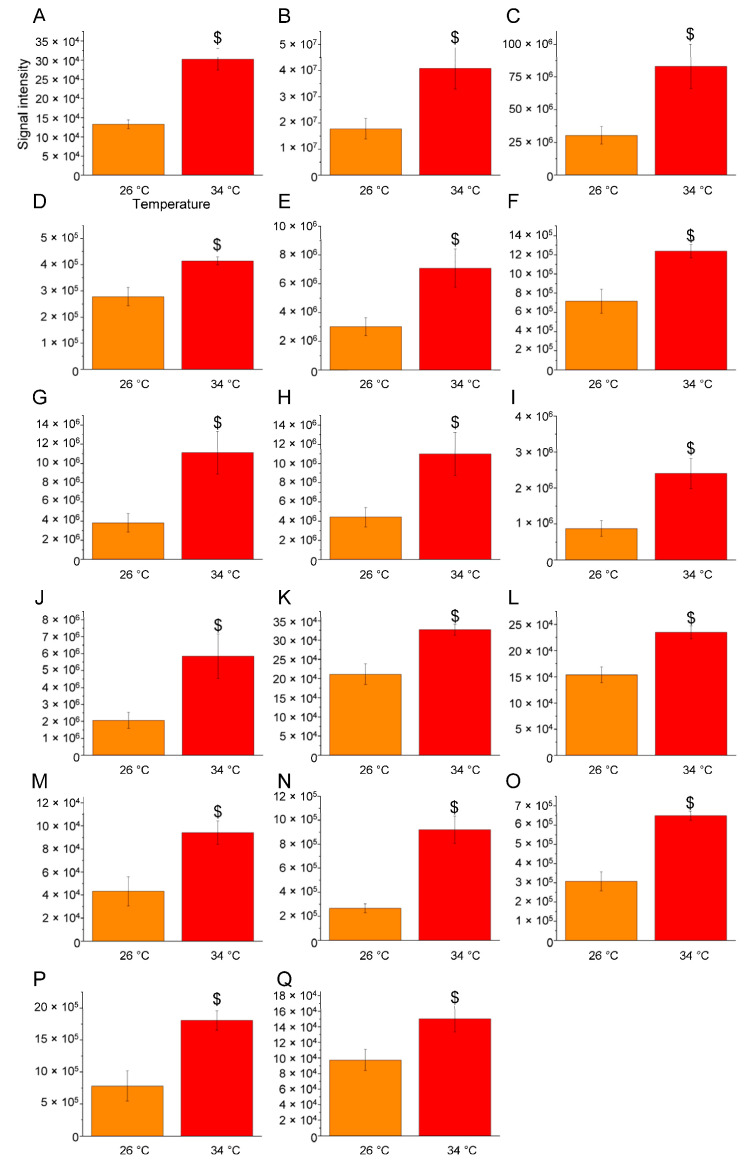
Lipids whose levels were significantly increased at 34 compared to 26 °C. Bar graphs display the relative amounts of brain lipids at 26 °C (orange) and 34 °C (red), focusing on those significantly elevated in the 34 °C vs. 26 °C comparison. Lipid species are as follows: (**A**): PE 34:0;O|PE 16:0_18:0;O (C39H78NO9P); (**B**): PE 34:1|PE 16:0_18:1 (C39H76NO8P); (**C**): PE 36:1|PE 18:0_18:1 (C41H80NO8P); (**D**): PE 38:3;O|PE 18:0_20:3;O (C43H80NO9P); (**E**): PE 40:1|PE 22:0_18:1 (C45H88NO8P); (**F**): PE 40:3;O|PE 18:0_22:3;O (C45H84NO9P); (**G**): PE 42:1|PE 24:0_18:1 (C47H92NO8P); (**H**): PE 42:2|PE 18:1_24:1 (C47H90NO8P); (**I**): PE 42:7|PE 18:1_24:6 (C47H80NO8P); (**J**): PE 44:1|PE 26:0_18:1 (C49H96NO8P); (**K**): LPE-N (FA)36:0|LPE-N (FA 18:0)18:0 (C41H82NO8P); (**L**): Cer 50:11;O4|Cer 28:4;O3 (FA 22:6) (C50H79NO5); (**M**): SM 33:0;O2 (C38H79N2O6P); (**N**): SM 34:1;O3 (C39H79N2O7P); (**O**): SM 38:6;O2|SM 17:2;O2/21:4 (C43H77N2O6P); (**P**): SM 44:0;O2|SM 18:0;O2/26:0 (C49H101N2O6P); (**Q**): SM 46:6;O3 (C51H93N2O7P). The data are expressed as means ± S.E.M. and were analysed via one-way ANOVA with Tukey’s post hoc correction. $ *p* ≤ 0.05 in 34 °C versus 26 °C comparison. *p*-values are reported in Table 2. *N* = 4.

**Figure 7:**
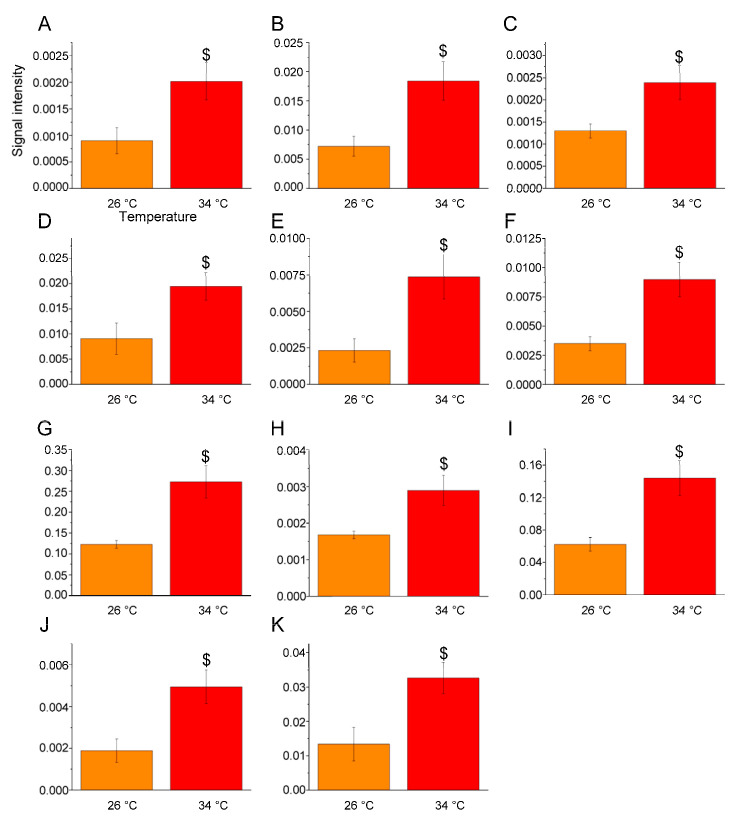
Gangliosides whose levels were significantly increased at 34 °C compared to 26 °C. Bar graphs display the relative amounts of brain gangliosides at 26 °C (orange) and 34 °C (red), focusing on those significantly elevated in the 34 °C vs. 26 °C comparison. Gangliosides species are as follows: (**A**): GD0a/b(36:0)-2H (C92H161N5O44); (**B**): GD1a/b (d18:1/18:0) (C85H149N3O39); (**C**): GD1a/b(36:1) (OH) (C84H148N4O40); (**D**): GD1a/b(38:1) (NeuGc)-2H/O-Ac-GD1b(36:0)-2H (C86H150N4O40); (**E**): GM2 d18:1-18:0 (C67H121N3O26); (**F**): GM3 d18:1-18:0 (C59H108N2O21); (**G**): GQ1a/b(36:0) (C106H182N6O55); (**H**): GQ1a/b(36:2) (C106H178N6O55); (**I**): GT3(36:0) (C81H142N4O37); (**J**): GT3(42:2) (C87H150N4O37); (**K**): O-Ac-GD1a/b(38:0)-2H/GD1a/b(40:2) (OH)-2H (C88H154N4O40). The data are expressed as means ± S.E.M. and were analysed via one-way ANOVA with Tukey’s post hoc correction. $ *p* ≤ 0.05 in 34 °C versus 26 °C comparison. *p*-values are reported in Table 2. *N* = 4.

**Figure 8:**
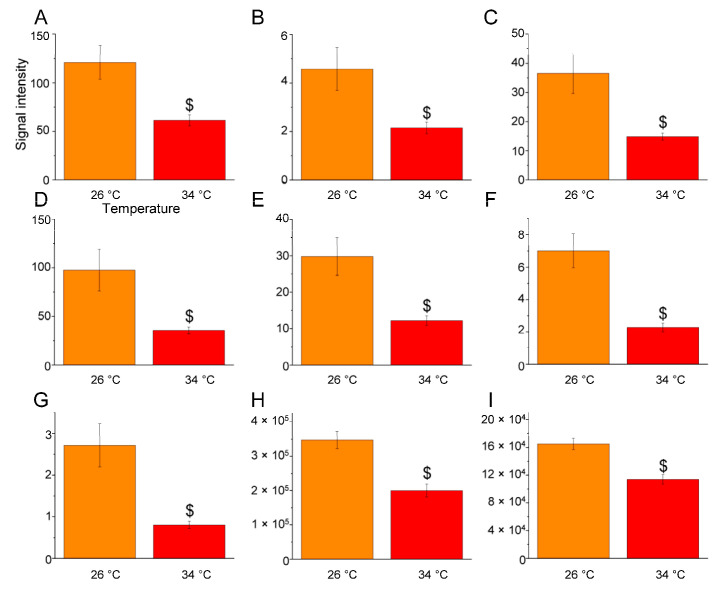
Lipids whose levels were significantly reduced at 34 °C compared to 26 °C. Bar graphs display the relative amounts of brain lipids at 26 °C (orange) and 34 °C (red), focusing on those significantly decreased in the 34 °C vs. 26 °C comparison. Lipid species include: (**A**): LPE O-18:2 (C23H46NO6P); (**B**): LPE O-18:3 (C23H44NO6P); (**C**): LPE O-19:1 (C24H50NO6P); (**D**): LPE O-20:1 (C25H52NO6P); (**E**): LPE O-20:2 (C25H50NO6P); (**F**): PE O-42:1|PE O-18:1_24:0 (C47H94NO7P); (**G**): PE O-44:1|PE O-18:1_26:0 (C49H98NO7P); (**H**): PG O-38:3|PG O-20:2_18:1 (C44H83O9P); (**I**): MGDG 34:1|MGDG 16:0_18:1 (C43H80O10). The data are expressed as means ± S.E.M. and were analysed via one-way ANOVA with Tukey’s post hoc correction. $ *p* ≤ 0.05 in $ 34 °C versus 26 °C comparison. *p*-values are reported in Table 2. *N* = 4.

In the zebrafish acclimatised to 34 °C, a broader alteration in lipid composition was observed compared to that observed for the zebrafish acclimatised to 26 °C (Table 2 and Figure 6, Figure 7 and Figure 8). Notably, phospholipids were altered, with an increase in levels of phosphatidylethanolamine (PE) molecules, including PE 34:0;O|PE 16:0_18:0;O (*p* < 0.0002), PE 34:1|PE 16:0_18:1 (*p* < 0.0397), PE 36:1|PE 18:0_18:1 (*p* < 0.0274), PE 38:3;O|PE 18:0_20:3;O (*p* < 0.0263), PE 40:1|PE 22:0_18:1 (*p* < 0.0482), PE 40:3;O|PE 18:0_22:3;O (*p* < 0.0208), PE 42:1|PE 24:0_18:1 (*p* < 0.0243), PE 42:2|PE 18:1_24:1 (*p* < 0.0452), PE 42:7|PE 18:1_24:6 (*p* < 0.0233), PE 44:1|PE 26:0_18:1 (*p* < 0.0384), and lysophosphatidylethanolamine LPE-N (FA)36:0|LPE-N (FA 18:0)18:0 (*p* < 0.0072) (Figure 6A–K).

High temperatures also led to an increase in the concentrations of certain sphingolipid molecules (Table 2 and Figure 6), including ceramides, (Cer) such as Cer 50:11;O4|Cer 28:4;O3 (FA 22:6) (*p* < 0.0034) (Figure 6K), and various sphingomyelins, like SM 33:0;O2 (*p* < 0.0103), SM 34:1;O3 (*p* < 0.0004), SM 38:6;O2|SM 17:2;O2/21:4 (*p* < 0.0002), SM 44:0;O2|SM 18:0;O2/26:0 (*p* < 0.0043), and SM 46:6;O3 (*p* < 0.0499) (Figure 6M–Q). The levels of gangliosides, such as GD0a/b(36:0)-2H (*p* < 0.0317); GD1a/b (d18:1/18:0) (*p* < 0.0131); GD1a/b(36:1) (OH) (*p* < 0.0314); GD1a/b(38:1) (NeuGc)-2H/O-Ac-GD1b(36:0)-2H (*p* < 0.0406); GM2 d18:1-18:0 (*p* < 0.0094); GM3 d18:1-18:0 (*p* < 0.0104); GQ1a/b(36:0) (*p* < 0.0099); GQ1a/b(36:2) (*p* < 0.0413); GT3(36:0) (*p* < 0.0093); GT3(42:2) (*p* < 0.0113); and O-Ac-GD1a/b(38:0)-2H/GD1a/b(40:2) (OH)-2H (*p* < 0.0168), were also increased (Figure 7A–K).

Conversely, a significant reduction in ether lipid levels was observed at 34 °C (Table 2 and Figure 8). The levels of ether lysophosphatidylethanolamine (LPE O) molecules, such as LPE O-18:2 (*p* < 0.0263), LPE O-18:3 (*p* < 0.0438), LPE O-19:1 (*p* < 0.0326), LPE O-20:1 (*p* < 0.0387), and LPE O-20:2 (*p* < 0.0283) (Figure 8A–E); ether phosphatidylethanolamines (PE O) like PE O-42:1|PE O-18:1_24:0 (*p* < 0.0058) and PE O-44:1|PE O-18:1_26:0 (*p* < 0.0107) (Figure 8F–G); and ether phosphatidylglycerols (PG O) such as PG O-38:3|PG O-20:2_18:1 (*p* < 0.0079) (Figure 8H) were reduced. Additionally, glycolipid monogalactosyldiacylglycerols (MGDG) such as MGDG 34:1|MGDG 16:0_18:1 (*p* < 0.0282) (Figure 8I) also showed a reduction at 34 °C.

## 3. Discussion

In recent years, studies have explored thermal variation and tolerance in fish models, including zebrafish. However, the mechanisms behind thermal tolerance limits remain inadequately understood [26,39,40,41,42,43]. It has been suggested that at the neuronal level, thermal tolerance is constrained by the loss of membrane integrity, while disruptions in neuronal function may also play a role [44]. Acute thermal exposure impacts fish through three main molecular mechanisms, namely, reaction rates, protein structure, and membrane fluidity [41], influencing enzyme activity, membrane protein function, mitochondrial performance, ROS generation, and ion balance [41].

Our group’s recent studies have revealed significant behavioural and brain proteome changes in zebrafish exposed to 18 °C, 26 °C, and 34 °C for 21 days (Figure 1) [19,20,22]. These temperatures, selected based on the zebrafish’s preferred thermal range (25–27 °C) [26], represent 8 °C deviations from the control, placing the fish at key points within their viable thermal range. According to previous findings, zebrafish acclimated to 26 °C have a cold and heat shock temperature (T_C_ and T_H_) of 14 °C and 38 °C, respectively. Therefore, our selected temperatures of 18 °C and 34 °C are 4 °C above T_C_ and 4 °C below T_H_, respectively, making them equidistant from the zebrafish’s viable thermal range boundaries.

The behavioural changes in warm-acclimatized and cold-acclimatized zebrafish observed in previous studies suggest altered nervous system function, and the results of proteomic analyses show that temperature affects not only the chemical–physical properties of proteins, such as flexibility, chemical bonds, substrate affinity, and catalytic rates, but also their expression levels [19,20,22,41].

This study extends the focus of research to the brain lipidome, recognising lipids’ crucial role in cellular and organelle membranes, affecting essential cellular processes.

**Figure 9:**
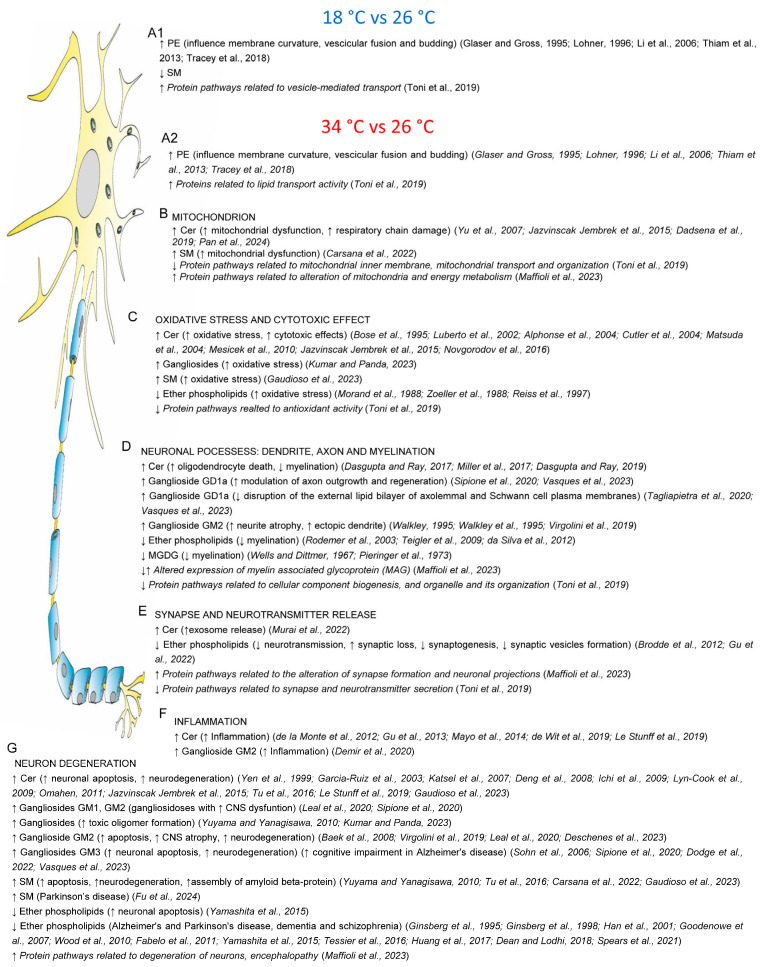
Schematic representation of the main alterations in the lipid component of the zebrafish brain caused by exposure to low (18 °C vs. 26 °C, above) and high (34 °C vs. 26 °C, below) temperatures and their possible effects. Literature suggests that the observed lipid alterations may impact several cellular and neurological processes, including membrane curvature, vesicular fusion, and budding (**A1**,**A2**), mitochondrial function (**B**), oxidative stress and cytotoxicity (**C**), neuronal structures such as dendrites, axons, and myelination (**D**), synaptic activity and neurotransmitter release (**E**), inflammation (**F**), and neurodegeneration (**G**). Downregulation (↓) and upregulation (↑) are indicated. Possible effects, deduced from information from the literature, are indicated in parentheses. Proteome alterations identified in previous work are reported in italics. Previous work are reported in italics: [19,45,46,47,48,49] (**A1**,**A2**), [19,21,51,52,53,54,55] (**B**), [19,51,56,57,58,59,60,61,62,63,64,65,66,67] (**C**), [19,21,68,69,70,71,72,73,74,75,76,77,78,79,80,81] (**D**), [19,21,82,83,84] (**E**), [85,86,87,88,89,90] (**F**), [21,51,55,63,64,71,72,76,88,91,92,93,94,95,96,97,98,99,100,101,102,103,104,105,106,107,108,109,110,111,112,113,114,115,116] (**G**).

Zebrafish exposed to 18 °C and 34 °C showed increased levels of specific phosphatidylethanolamine molecules, which are known to influence membrane curvature [45] and fluidity [46] and modulate crucial neuronal processes like vesicular budding and membrane fusion [47,48,49] (Figure 9A1,A2). This finding aligns with previous observations for teleost fish, such as Crucian carp (*Carassius carassius*), wherein an increase in phosphatidylethanolamine levels serves as a lipid response induced to maintain membrane fluidity at low temperatures [50]. The lipid alteration observed in zebrafish at both low and high temperatures is consistent with increased expression of proteins involved in vesicle-mediated transport at 18 °C and lipid transporter activity at 34 °C [19].

Conversely, the levels of specific sphingomyelin molecules decreased at 18 °C and increased at 34 °C. Elevated sphingomyelin levels, associated with neurodegenerative conditions like Parkinson’s disease, contribute to oxidative stress and neurodegeneration [64,105]. The levels of other sphingolipids, such as ceramide and gangliosides, also varied with temperature, with gangliosides playing a key role in the thermal adaptation of neuronal membranes [50,117,118,119,120].

In our study, a targeted lipid analysis focused on gangliosides was conducted alongside an untargeted lipid analysis. While studies on fish adapted to different temperatures or subjected to thermal changes have highlighted a general rule regarding ganglioside composition during thermal adaptation (wherein lower environmental temperatures lead to more polar brain gangliosides [50]), no significant alterations in ganglioside levels were observed at 18 °C compared to 26 °C. This could be due to the 21-day thermal treatment being insufficiently long or the temperature variation not being substantial enough. Conversely, a significant increase in levels of ganglioside molecules was observed in the brains of fish exposed to 34 °C, suggesting that high temperatures impact lipid composition more than low temperatures under our experimental conditions.

The increase in the levels of ceramide, ganglioside, and sphingomyelin molecules at 34 °C can significantly affect the nervous system, as sphingolipids are involved in crucial functions such as membrane organisation [121,122,123,124,125], neuronal differentiation [126,127], cell adhesion [128], signal transduction [129], inflammation [130], neurite outgrowth [127,131], neuronal-glial interactions, myelin stability [132], membrane-channel activity [133], and the regulation of neurotransmitter receptor conformation and function [134].

The lipid changes observed at 34 °C suggest potential neuronal and glial cell stress, also implying mitochondrial dysfunction and increased susceptibility to oxidative stress in neurons. Studies have linked increased ceramide and sphingomyelin levels observed at 34 °C with mitochondrial dysfunction, reactive oxygen species production, and altered mitochondrial outer-membrane permeability [51,52,53,54,55] (Figure 9B). These changes can lead to abnormal energy metabolism, impaired mitophagy, defective kinetics and transport [52], and disrupted protein homeostasis [135]. Mitochondrial alterations align with proteomic findings showing modified expression of proteins involved in mitochondria and energy metabolism in zebrafish at 34 °C [19,21]. Interestingly, mitochondrial functions are among the main pathways through which acute thermal exposure impacts fish [41], and mitochondria are key markers of neurotoxicity in zebrafish [18].

The cytotoxic effect of high temperatures and their ability to generate oxidative stress in CNS cells are also consistent with the increase in ganglioside levels [51,56,57,58,59,60,61,62,63,64] and the reduction in ether phospholipid levels at 34 °C [65,66,67] (Figure 9C). This cytotoxicity may be exacerbated by decreased levels of proteins associated with antioxidant activity noted in previous studies [19]. Gangliosides, which are sialic-acid-containing glycosphingolipids primarily located in the outer leaflets of plasma membranes of nervous system cells [125], play a critical role in CNS homeostasis, with both deficiency and excess linked to severe neurodegenerative conditions such as Alzheimer’s disease, Parkinson’s disease, and amyotrophic lateral sclerosis, as well as injury conditions [71,72]. Ether lipids are involved in key processes such as membrane fusion, the maintenance of lipid raft microdomain stability, cell differentiation, and signalling pathway activity [108] and may reduce oxidative stress due to their potential role as endogenous antioxidants [65,66,67]. Notably, altered ROS generation is one of the main pathways through which acute thermal exposure impacts fish [41], with oxidative stress being a primary marker of neurotoxicity analysed in zebrafish [18].

The altered lipid composition at 34 °C could affect dendrite, axon, and myelin sheath characteristics (Figure 9D). Increases in ceramide levels, reductions in ether phospholipid levels, and decreases in monogalactosyl diglyceride levels (MGDGs) at 34 °C have been linked to oligodendrocyte death and reduced myelination [68,69,70]. Interestingly, proteomic analyses have detected altered expression of myelin-associated glycoprotein (MAG) in the brains of zebrafish maintained at 34 °C [21]. MGDGs, a class of neutral glycerolipids [136] present in small amounts in the mammalian nervous system [80,137,138,139], have been proposed as markers of myelin and myelination [80,138], with their highest biosynthesis rate and levels occurring during peak myelin production in postnatal development [80,81]. Additionally, the increase in ganglioside GM2 levels at 34 °C has been associated with neurite atrophy and ectopic dendrite formation [74,75,76]. Further information and bibliographic references are summarised in Figure 9 [72,73,77,78,79]. These results, combined with the reduction in the activity of protein pathways related to cellular component biogenesis [19], suggest that high temperatures may impair nerve impulse transmission along the axon. Notably, neurons’ morphology and characteristics are key markers of neurotoxicity in zebrafish [18].

The findings indicate that high temperatures can lead to synaptic alteration and potential malfunction (Figure 9E). The increase in ceramide levels at 34 °C was associated with increased exosomal release [82], while the reduction in levels of ether phospholipids was linked to diminished synaptic function [83,84]. Furthermore, proteomic analyses highlighted an increase in protein pathways related to the alteration of synapse formation and neuronal projections [21], along with a reduction in those involved in synapse and neurotransmitter secretion [19]. Alterations in neurotransmission are key markers of neurotoxicity in zebrafish [18].

The rise in temperature could also promote neuroinflammation, as evidence shows that elevated ceramide and ganglioside GM2 can stimulate the expression of pro-inflammatory molecules [85,86,87,88,89,90] (Figure 9F).

The altered lipid profile and proteome observed in the zebrafish brains at 34 °C suggest a potential condition of neuronal degeneration (Figure 9G). The increase in the levels of ceramide, GM2 and GM3 gangliosides, and sphingomyelin, coupled with the reduction in the levels of ether phospholipids at 34 °C, were associated with increased apoptosis and neuronal death [51,55,64,71,72,76,88,91,92,93,94,95,96,97,98,99,100,101,102,103,104,106] and neurodegenerative diseases such as Alzheimer’s and Parkinson’s. [71,72,103,104,105,106,107,108,109,110,111,112,113,114,115,116]. This scenario is compatible with the increase in protein pathways associated with neuronal degeneration and encephalopathy detected at the same temperature [19].

In our experimental model, the effects of warm acclimatisation on brain lipid composition appear to be more pronounced than those of cold acclimatisation, as indicated by the greater number of altered lipid molecules at 34 °C compared to 18 °C. This may be due to the specific temperature settings used in our study. In fact, it has been demonstrated that T_C_ and T_H_ vary depending on the acclimation temperature (T_A_), stabilising after a period of at least six days. Based on Aslanidi and Kharakoz’s findings [26], the adapted T_C_ for zebrafish acclimated to 18 °C is approximately 8 °C, while the adapted T_H_ for those acclimated to 34 °C is around 42 °C. This indicates that the warm-acclimatised zebrafish in our study were exposed to a temperature 8 °C below their adapted T_H_ that corresponds to the upper boundary of the zebrafish’s viable thermal range, whereas the cold-acclimatised zebrafish experienced a temperature 10 °C above their adapted T_C_ that corresponds to the lower boundary of the zebrafish’s viable thermal range. Further studies involving zebrafish acclimatised to temperatures below 18 °C and above 34 °C could offer further insights into the impact of extreme temperatures on brain lipid composition.

## 4. Materials and Methods

### 4.1. Fish Husbandry and Experimental Setup

A total of 120 adult (6–7 months old) wild-type zebrafish, with an approximate sex ratio of 50:50 (male to female), were used in this study. Zebrafish husbandry and thermal treatments were performed as previously reported [19] and are briefly described below. At the beginning of the study, the fish were randomly divided equally into three tanks (40 × 30 × 30 cm, width × depth × height) and kept at 26 °C for 10 days to allow them to acclimate. To ensure optimal water quality, each tank was equipped with external filter systems (Eden 511 h, Eden s.r.l., Vicenza, Italy) maintaining a constant flow of filtered water (600 L/h). Additionally, the water was continuously aerated by aquarium aerators (Sicce AIRlight, SICCE s.r.l, Vicenza, Italy, 3300 cc/min 200 L/h). Water temperature was regulated using digital thermostats (Eden 430, Eden s.r.l., Vicenza, Italy) connected to heating coils (Eden 415, 230 V, 50/60 Hz, 80 W, Eden s.r.l., Vicenza, Italy) supplemented by a cooling system and monitored daily with hand thermometers. The chemical and physical characteristics of the tank water were checked at least twice per week using a Sera Aqua-test Box Kit (Sera GmbH, Heinsberg, Germany) and an eSHa Aqua Quick Test (eSHa Lab, Maastricht, The Netherlands). No differences were found between the treatment and control tanks. Total ammonia (NH3/NH4) and nitrite (NO_2_) levels were not detected (i.e., they remained below the detection limit of 0.05 mg/L), and the water was partially renewed whenever the nitrate concentration reached 25 mg/L. The water pH was 7, the conductivity ranged between 400 and 500 micro-Siemens, the degrees of total hardness ranged between 7 and 14, and the degree of carbonate hardness (dKH) was 6. The phosphate (PO_4_) levels were <1 mg/L, and no copper (Cu) or chlorine (Cl_2_) was detected. No significant difference was found between the tanks.

Faeces and food waste were removed from the tanks at least three times per week. During cleaning, a water exchange of approximately 20–30% was performed weekly to maintain the correct water volume and preserve chemical–physical parameters. The fish were maintained under an artificial photoperiod (12:12 light/dark cycle) and fed three times daily (10 am, 2 pm, and 6 pm) with commercial dry granular food (TropiGranMIX, Dajana per sro, Bohuňovice, Czech Republic) using automatic feeders (Eden 90, Eden s.r.l., Vicenza, Italy). Food (0.6 g/day) was administered to each tank, allowing zebrafish at the three temperatures to feed themselves according to their appetites.

After the 10-day acclimation period, one of the three tanks was randomly assigned to be the 18 °C tank, and two others were assigned to be the 26 °C (control) and 34 °C tanks. The temperatures of the respective tanks were gradually adjusted to 18 ± 1 °C, 26 ± 1 °C (control), and 34 ± 1 °C over 72 h, setting a variation of ±2 °C every 16 h.

The fish were then maintained at these temperatures for 21 days. No fish died during thermal treatment.

At the end of the tests, fish were euthanised via prolonged immersion in a solution of tricaine methanesulfonate (MS-222, 300 mg/L). Post-euthanasia, brains were dissected for lipidomic analyses.

### 4.2. Lipidomic Analyses

Forty brains for each temperature were pooled in 4 groups (10 brains/group), homogenised via sonication, and centrifuged. The protein content of the supernatant was determined using a NanoDrop spectrophotometer, and each specimen was treated as follows. In total, 5 µL of IS1 (Splash Lipidomix Internal Standards, Avanti Polar, Lipids Inc., Alabaster, AL, USA), 5 µL of IS2 (Hippuric acid 13C6 (0.5 mg/mL) in methanol), and 1 mL of dichloromethane/methanol (2:1, *v*/*v*) were added to the sample. After shaking and sonication (15 min), centrifugal separation (15 min, 13,000 rpm, 15 °C) was employed to separate the aqueous and organic phases.

The organic phase was used for untargeted lipidomics analysis. This phase was dried under a nitrogen flow, and the obtained residue was resuspended with 50 µL of 2-propanol/acetonitrile (90:10, *v*:*v*), 0.1% formic acid, and 10 mM of ammonium acetate (FMB). All samples were further diluted in a 1:10 ratio (*v*:*v*) with FMB before being analysed. 

The aqueous phase was used for the targeted analysis aimed at searching for gangliosides. It was filtered through 0.2 µm RC filters and dried in Speedvac, and the obtained residue was resuspended with 50 µL of methanol. 

Moreover, a “Blank” sample, to which no IS was added, and a “Pool” sample, to which 10 μL of each sample was added, were also prepared and analysed in both untargeted and targeted lipidomics analyses. 

All samples for untargeted and targeted lipidomics analysis were analysed using Agilent 1290 Infinity II LC (Agilent, Technologies, Santa Clara, CA, USA) coupled with a ZenoTOF 7600 System (SCIEX, Framingham, MA, USA) equipped with Turbo V™ Ion Source with ESI Probe. 

In the untargeted lipidomics analysis, chromatographic separation was achieved using a Kinetex^®^ EVO C18 (Phenomenex^®^, Torrance, CA, USA) (1.7 µm, 100 × 2.1 mm), equipped with a pre-column, with an elution gradient from 55% of mobile phase A (Ammonium acetate 10 mM in Water/Acetonitrile (60:40 *v*/*v*), 0.1% formic acid) to 97% of mobile phase B (Ammonium acetate 10 mM in 2-propanol/Acetonitrile (90:10 *v*/*v*), 0.1% formic acid), in 10 min, with a total run of 20 min and at a flow rate of 0.400 mL/min. The column and auto-sampler temperatures were set to 45 °C and 15 °C, respectively. The sample injection volume was 5 µL. Two replicates for each sample were analysed. Nitrogen was used as a nebulizing gas (GS1, 55 psi), turbo spray gas (GS2, 65 psi), curtain gas (CUR, 35 psi), and CAD gas 7, and source temperature was 500 °C. Spray Voltage was fixed at 4.5 kV (−4.5 kV in negative mode), declustering potential (DP) was 80 eV, and the collision energy was 35 eV, with a collision energy spread (CES) of 15 eV. MS spectra were collected over an *m*/*z* range of 140–1500 Da in full-mass scan (250 ms accumulation time), operating in IDA^®^ mode (Information-Dependent Acquisition) from 50 to 1500 Da (50 ms accumulation time, top-20 spectra). Data analysis was carried out using MS-DIAL (ver. 5.1) with the integrated LipidBlast database (version 68) [140].

Samples for the targeted lipidomics analysis were separated using a Kinetex^®^ HILIC (Phenomenex, Torrance, CA, USA) (1.7 µm, 100 × 2.1 mm) equipped with a pre-column. The temperature was set at 45 °C. The analytes were eluted in gradient from 100% buffer B (ammonium acetate (10 mM) in water/acetonitrile (10:90, *v*:*v*)) to 50% buffer A (ammonium acetate (10 mM) in water/acetonitrile (90:10, *v*:*v*)) in 8.5 min, with a total run time of 20 min at a constant flow rate of 0.400 mL/min. All samples were analysed in duplicate and in negative mode with electrospray ionization, and the injection volume was 5 µL. The instrument settings were as follows: GS1 = 55 psi, GS2 = 65 psi, CUR = 35 psi, CAR = 7, spray voltage −4.0 kV, and a source temperature of 375 °C. MS spectra were acquired via a full-mass scan from 150 to 2000 Da (250 ms accumulation time), operating in IDA^®^ mode (Information Dependent Acquisition) from 75 to 2000 Da (50 ms accumulation time, top-20 spectra), with a declustering potential (DP) of 120 eV and a collision energy of 70 V with a collision energy spread (CES) of 30. Data processing was carried out using SCIEX OS software (ver. 3.3.1) (SCIEX™, Framingham, MA, USA) integrated with the Formula Finder algorithm that predicts potential chemical formulae based on the MS and MS/MS spectra by using the precursor ion’s mass accuracy, isotopic pattern, and MS/MS fragmentation pattern.

Untargeted data analysis was carried out using MS-DIAL (ver. 5.1) with the integrated LipidBlast database (version 68). Briefly, among the number of identifications (ID), based on the *m*/*z* (parent ion) value, those considered positively identified had a CV% lower than 30%, in the “Pool” samples only, and a “Known” identification attributed by the software. For these IDs, the correspondence between the acquired MS/MS spectrum and that present in the LipidBlast database was verified. For each ID identified, the corresponding area of each injection was extracted, and the averages were calculated (*n* = 2).

Targeted data processing was carried out using SCIEX OS ver 3.3.1 (SCIEX™, Concord, ON, Canada) software integrated with Formula Finder. For each identified peak, the corresponding area was extracted, and the area/IS area ratio and the average of the two injections were calculated.

### 4.3. Statistical Analysis

Statistical analysis was performed using Perseus software (version 1.5.5.3) [141]. Only the lipids present and quantified in at least 75% of the repeats were positively identified in a sample and used for statistical analysis (Tables S1 and S4). Lipids were considered to be differentially expressed if they were present only in one condition or showed significant *t*-test differences (Student’s *t*-test *p*-value ≤ 0.05) (Tables S2, S4 and S5). One-way ANOVA was performed on single lipid molecules across the three temperature conditions, followed by Tukey’s post hoc test. Differences were considered statistically significant at *p* ≤ 0.05 (Figure 4, Figure 5, Figure 6, Figure 7 and Figure 8 and Table 1 and Table 2).

For the analysis of lipid profiles shown in Figure 3, the results reported in Table S1 were analysed via two-way ANOVA with respect to the factors of temperature and number of double bonds in the fatty acid chains or temperature and number of carbon atoms in the fatty acid chains. Tukey’s post hoc test was used, and differences were considered statistically significant at *p* ≤ 0.05 (Figure 3). ANOVA statistical analyses were performed using Origin Pro 2018 (version 95E; OriginLab Corporation, Northampton, MA, USA) and R Studio (version 2023.12.1+402; RStudio Team, Boston, MA, USA) software. Results were expressed as the mean ± SEM.

### 4.4. Figure Preparation

CorelDRAW^®^ X7 (Version 17.0.0.491; Corel Corporation, Ottawa, ON, Canada) and Microsoft Power Point 365 (Microsoft Corporation, Redmond, WA, USA).were used to design the figures. Figure 1 was generated using Biorender (https://biorender.com, accessed on 14 August 2024).

## 5. Conclusions

The analysis of the brain lipid composition in adult zebrafish maintained at 18 °C, 26 °C, and 34 °C for 21 days reveals that both cold and warm acclimatisation significantly alter the relative abundance of specific lipid molecules. These findings suggest that the behavioural changes observed in zebrafish exposed to 18 °C or 34 °C are not solely attributable to altered protein expression but also involve modifications in lipid composition. Future research should focus on targeted lipidomic analyses to explore temperature-induced alterations in the membranes of specific cellular organelles, such as mitochondria.

## Figures and Tables

**Table 1:** Significantly altered lipid molecules in the comparison of 18 °C with 26 °C. Up- or downregulation, lipid categories, metabolite names, chemical formulas, and *p*-values are shown, and the corresponding graphs are shown in the figures cited in the last column.

			18 °C vs. 26 °C			
			Metabolite Name	Chemical Formula	*p* Value	Figure
Upregulated	Phospholipids	Phosphatidylethanolamine	PE 36:3;O|PE 16:0_20:3;O	C41H76NO9P	0.0437	Figure 4A
			PE 38:3;O|PE 18:0_20:3;O	C43H80NO9P	0.0152	Figure 4B
			PE 40:4;O|PE 18:1_22:3;O	C45H82NO9P	0.0040	Figure 4C
			PE 42:9;O|PE 22:6_20:3;O	C47H76NO9P	0.0184	Figure 4D
			PE 44:9;O|PE 22:6_22:3;O	C49H80NO9P	0.0230	Figure 4E
	Sphingolipids	Sphingomyelin	SM 40:7;O3	C45H79N2O7P	0.0101	Figure 4F
Downregulated	Sphingolipids	Sphingomyelin	SM 37:3;O2|SM 19:3;O2/18:0	C42H81N2O6P	0.0248	Figure 5A
			SM 38:1;O2|SM 21:0;O2/17:1	C43H87N2O6P	0.0253	Figure 5B
			SM 40:1;O2|SM 18:1;O2/22:0	C45H91N2O6P	0.0442	Figure 5C
			SM 40:2;O2|SM 18:1;O2/22:1	C45H89N2O6P	0.0071	Figure 5D
			SM 42:1;O3	C47H95N2O7P	0.0219	Figure 5E
			SM 42:2;O2|SM 18:1;O2/24:1	C47H93N2O6P	0.0188	Figure 5F
			SM 42:3;O2|SM 18:1;O2/24:2	C47H91N2O6P	0.0144	Figure 5G
			SM 42:4;O2|SM 18:1;O2/24:3	C47H89N2O6P	0.0216	Figure 5H
			SM 44:3;O2|SM 18:1;O2/26:2	C49H95N2O6P	0.0256	Figure 5I
			SM 44:4;O2|SM 18:1;O2/26:3	C49H93N2O6P	0.0497	Figure 5J

**Table 2:** Significantly altered lipid molecules in the 34 °C vs. 26 °C comparison. Up- or downregulation, lipid categories, metabolite names, chemical formulas, and *p*-values are shown, and the corresponding graphs are shown in the figures cited in the last column.

			34 °C vs. 26 °C			
			Metabolite Name	Chemical Formula	*p* Value	Figure
Upregulated	Phospholipids	Phosphatidylethanolamine	PE 34:0;O|PE 16:0_18:0;O	C39H78NO9P	0.00024848	Figure 6A
			PE 34:1|PE 16:0_18:1	C39H76NO8P	0.0397	Figure 6B
			PE 36:1|PE 18:0_18:1	C41H80NO8P	0.0274	Figure 6C
			PE 38:3;O|PE 18:0_20:3;O	C43H80NO9P	0.02629	Figure 6D
			PE 40:1|PE 22:0_18:1	C45H88NO8P	0.04822	Figure 6E
			PE 40:3;O|PE 18:0_22:3;O	C45H84NO9P	0.02079	Figure 6F
			PE 42:1|PE 24:0_18:1	C47H92NO8P	0.02425	Figure 6G
			PE 42:2|PE 18:1_24:1	C47H90NO8P	0.04518	Figure 6H
			PE 42:7|PE 18:1_24:6	C47H80NO8P	0.02326	Figure 6I
			PE 44:1|PE 26:0_18:1	C49H96NO8P	0.03839	Figure 6J
		Lyso phosphatidylethanolamine	LPE-N (FA)36:0|LPE-N (FA 18:0)18:0	C41H82NO8P	0.00716	Figure 6K
	Sphingolipids	Ceramide	Cer 50:11;O4|Cer 28:4;O3 (FA 22:6)	C50H79NO5	0.00344	Figure 6L
		Gangliosides	GD0a/b (36:0)-2H	C92H161N5O44	0.03171	Figure 7A
			GD1a/b (d18:1/18:0)	C85H149N3O39	0.0131	Figure 7B
			GD1a/b (36:1) (OH)	C84H148N4O40	0.03135	Figure 7C
			GD1a/b(38:1) (NeuGc)-2H/O-Ac-GD1b (36:0)-2H	C86H150N4O40	0.04056	Figure 7D
			GM2 d18:1-18:0	C67H121N3O26	0.00937	Figure 7E
			GM3 d18:1-18:0	C59H108N2O21	0.01035	Figure 7F
			GQ1a/b (36:0)	C106H182N6O55	0.00991	Figure 7G
			GQ1a/b (36:2)	C106H178N6O55	0.0413	Figure 7H
			GT3 (36:0)	C81H142N4O37	0.00928	Figure 7I
			GT3 (42:2)	C87H150N4O37	0.01129	Figure 7J
			O-Ac-GD1a/b (38:0)-2H/GD1a/b (40:2) (OH)-2H	C88H154N4O40	0.0168	Figure 7K
		Sphingomyelin	SM 33:0;O2	C38H79N2O6P	0.01027	Figure 6M
			SM 34:1;O3	C39H79N2O7P	0.00042476	Figure 6N
			SM 38:6;O2|SM 17:2;O2/21:4	C43H77N2O6P	0.0001563	Figure 6O
			SM 44:0;O2|SM 18:0;O2/26:0	C49H101N2O6P	0.00431	Figure 6P
			SM 46:6;O3	C51H93N2O7P	0.04989	Figure 6Q
Downregulated	Ether phospholipids	Lysophosphatidylethanolamine ether	LPE O-18:2	C23H46NO6P	0.02631	Figure 8A
			LPE O-18:3	C23H44NO6P	0.04381	Figure 8B
			LPE O-19:1	C24H50NO6P	0.03261	Figure 8C
			LPE O-20:1	C25H52NO6P	0.03871	Figure 8D
			LPE O-20:2	C25H50NO6P	0.02834	Figure 8E
		Phosphatidylethanolamine ether	PE O-42:1|PE O-18:1_24:0	C47H94NO7P	0.00582	Figure 8F
			PE O-44:1|PE O-18:1_26:0	C49H98NO7P	0.01071	Figure 8G
		Phosphatidylglycerol ether	PG O-38:3|PG O-20:2_18:1	C44H83O9P	0.00793	Figure 8H
	Glycolipids	Monogalactosyldiacylglycerol	MGDG 34:1|MGDG 16:0_18:1	C43H80O10	0.02816	Figure 8I

## Data Availability

Dataset available on request from the authors.

## Supplementary Materials

The following supporting information can be downloaded at https://www.mdpi.com/article/10.3390/ijms25179629/s1.
